# Influence of Examiner Experience on the Measurement of Bone-Loss by Low-Dose Cone-Beam Computed Tomography: An Ex Vivo Study

**DOI:** 10.3390/jimaging10080177

**Published:** 2024-07-23

**Authors:** Maurice Ruetters, Korallia Alexandrou, Antonio Ciardo, Sinclair Awounvo, Holger Gehrig, Ti-Sun Kim, Christopher J. Lux, Sinan Sen

**Affiliations:** 1 Department of Operative Dentistry, University Hospital Heidelberg, Im Neuenheimer Feld 400, 69120 Heidelberg, Germanyholger.gehrig@med.uni-heidelberg.de (H.G.); ti-sun.kim@med.uni-heidelberg.de (T.-S.K.); 2 Department of Orthodontics, University Hospital Heidelberg, Im Neuenheimer Feld 400, 69120 Heidelberg, Germany; korallia.alexandrou@med.uni-heidelberg.de (K.A.); christopher.lux@med.uni-heidelberg.de (C.J.L.); 3 Institute of Medical Biometry, University Hospital Heidelberg, Im Neuenheimer Feld 130.3, 69120 Heidelberg, Germany; awounvo@imbi.uni-heidelberg.de; 4 Department of Orthodontics, University Hospital Schleswig Holstein, Arnold-Heller-Straße 3, 24105 Kiel, Germany; sinan.sen@uksh.de

**Keywords:** cone-beam computed tomography, low-dose cone-beam computed tomography, computed radiography, periodontitis, investigator experience

## Abstract

The aim of this study was to investigate the influence of examiner experience on measurements of bone-loss using high-dose (HD) and low-dose (LD) CBCT. Three diagnosticians with varying levels of CBCT interpretation experience measured bone-loss from CBCT scans of three cadaveric heads at 30 sites, conducting measurements twice. Between the first and second measurements, diagnostician 2 and diagnostician 3 received training in LD-CBCT diagnostics. The diagnosticians also classified the certainty of their measurements using a three-grade scale. The accuracy of bone-loss measurements was assessed using the absolute difference between observed and clinical measurements and compared among diagnosticians with different experience levels for both HD and LD-CBCT. At baseline, there was a significant difference in measurement accuracy between diagnostician 1 and diagnostician 2, and between diagnostician 1 and diagnostician 3, but not between diagnostician 2 and diagnostician 3. Training improved the accuracy of both HD-CBCT and LD-CBCT measurements in diagnostician 2, and of LD-CBCT measurements in diagnostician 3. Regarding measurement certainty, there was a significant difference among diagnosticians before training. Training enhanced the certainty for diagnosticians 2 and 3, with a significant improvement noted only for diagnostician 3. Examiner experience level significantly impacts the accuracy and certainty of bone-loss measurements using HD- and LD-CBCT.

## 1. Introduction

Three-dimensional imaging with cone-beam computed tomography (CBCT) has overcome the limitations of two-dimensional radiographs, such as geometric distortions and superimpositions from anatomical structures [1]. One limitation of routine CBCT use in daily dental practice is the high radiation dose compared with other imaging methods such as panoramic or periapical radiography [2,3,4].

To reduce the radiation dose, low-dose CBCT (LD-CBCT) protocols have been developed. These protocols can acquire three-dimensional images with similar radiation doses as required for a panoramic, lateral cephalometric, or full-mouth radiograph [2,4]. Ludlow et al. reported radiation doses of 12–29 µSv with these low-dose protocols [4]. This makes LD-CBCT an interesting alternative to established imaging techniques as it can show periodontic structures in three dimensions without the limitation of high radiation exposure.

Studies have shown that LD-CBCT can reliably depict the buccal alveolar bone with high precision. This detailed imaging capability is particularly beneficial in clinical practice, as it helps clinicians avoid undesirable side effects such as gingival recessions following orthodontic treatment, thus enhancing patient outcomes [5,6,7,8]. Further investigations have demonstrated that LD-CBCT is also effective in accurately depicting peri-implant defects, providing valuable diagnostic information for implantology [9]. However, it is important to note that in these studies, CBCT analyses were performed by highly experienced and trained investigators, which could influence the outcomes [7,8,10]. The level of examiner experience has been shown to significantly affect the accuracy of high-dose (HD) CBCT reporting in endodontology, suggesting that training and experience are crucial factors in obtaining reliable and accurate diagnostic results [11]. Especially in endodontics, three-dimensional imaging is superior to two-dimensional imaging and greatly facilitates diagnostics [12].

In the present study, our research objectives were to determine the extent to which examiner experience affects the accuracy of buccal-lamella measurements made using HD-CBCT and LD-CBCT, and to evaluate the effect of a single training session on measurement accuracy. Our hypotheses are that examiner experience influences the accuracy of measurements of the buccal lamella in HD-CBCT and LD-CBCT and that a single training session improves the accuracy and certainty of measurements in HD-CBCT and LD-CBCT.

## 2. Materials and Methods

Data were collected from three human cadaveric heads which had been cut in half sagittally. The cadavers were preserved in 99% ethanol, glycerin, and 37% formalin. Measurements were taken from 20 teeth at 30 default sites. The specimens were the heads of body donors the Department of Anatomy and Cell Biology of the University of Heidelberg. The anatomical body donors had already given their consent beforehand for the donation of their bodies to the Department of Anatomy and Cell Biology of the University of Heidelberg for research purposes. A HD-CBCT and LD-CBCT scan was taken at each site. At the time of the radiographic investigation, the human heads included mandibles that were fully covered by soft tissue and adjacent cheek muscles. The tongue, neck muscles, skull base, and cervical vertebrae were also still present.

### 2.1. Cone-Beam Computed Tomography

During the volumetric recordings, the heads were fixed with their throat in a tube and orientated according to the manufacturer’s orientation lines. Imaging was performed on a CBCT scanner (Orthophos 3D SL ^®^, DentsplySirona, Bensheim, Germany). The volumetric acquisition conditions for HD-CBCT images were as follows:

Radiation exposure time, 14.2 s; 6 mA; 85 kV; field of view, 8 × 8 cm^2^; isotropic voxel size, 0.16 mm; dose area product (DAP), 943 mGy cm^2^.

The acquisition conditions for LD-CBCT scans were as follows:

Radiation time, 2.1 s; 10 mA; 85 kV; field of view, 8 × 8 cm^2^; isotropic voxel size, 0.16 mm; DAP, 67 mGy cm^2^.

### 2.2. Reference Measurements

**Figure 1:**
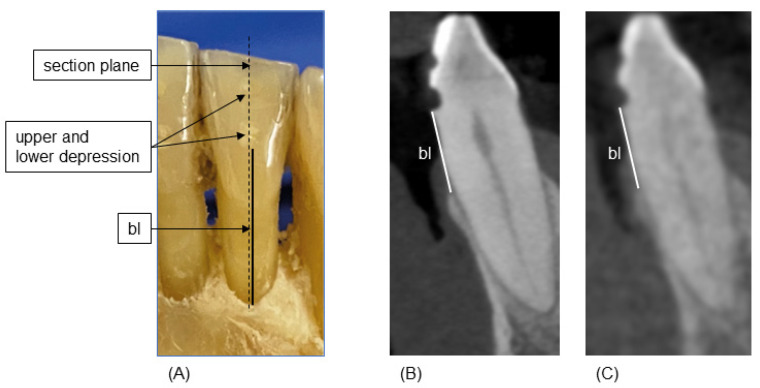
Measurement scheme. (**A**): reference measurements of buccal bone height (bl) from the most apical point of the lower depression to the most coronal point of the buccal bone (solid black line). (**B**,**C**): corresponding sagittal planes of HD-CBCT (**B**) and LD-CBCT (**C**). White lines indicate the bl.

Reference measurements were collected as previously described [7]. Briefly, after radiological imaging, the gingiva were carefully removed using microsurgical instruments to ensure the buccal and oral bone were not damaged. Then, in the axis of the previously milled depressions, the distance from the most apical point of the lower depression to the alveolar crest was measured on the buccal and oral aspect of the investigated teeth using a 0.1 mm periodontal probe (Florida Probe, Clark Dental Equipment Systems Ltd., Cheshire, UK). This established a reference standard for buccal bone height (bl) measurements (Figure 1A). These measurements were made by one experienced investigator (M.R.), who had previously been calibrated on a model. For calibration, the investigator had to successfully reproduce (i.e., a relative agreement of 95%) the principal investigator’s (T.K.) bone-sounding measurements of clinical attachment loss at 168 sites on a standardized ex vivo reference model with transparent gingiva (Co. M. Tech, Seoul, Republic of Korea).

### 2.3. Image Review

Images were reviewed as previously described [7]. Briefly, data from HD-CBCT and LD-CBCT scans were exported in DICOM format into the application software OSIRIX pro (aycanOsiriX, version 2.06.000). Contrast, magnification, and volume orientation could be modified, and scrolling through the volume in three-dimensional multiplanar reformations was also allowed. Images were evaluated on the same workstation and monitor (iMac, 27-inch, Apple, Cupertino, CA, USA) in the same dark room.

The images were reviewed by three dentists (M.R., T.C., and K.A.) in multiplanar reconstruction. Diagnostician 1 had more than eight years of experience in HD-CBCT diagnostics and more than three years in LD-CBCT diagnostics. Diagnostician 2 had 4 years of experience in HD-CBCT diagnostics and no experience in LD-CBCT diagnostics. Diagnostician 3 had 6 months of experience in HD-CBCT diagnostics and no experience in LD-CBCT diagnostics. For the orientation procedure, two depressions were identified and the axis of the coronal plane was placed through the center of the depressions. The axial slice was then aligned with the lower depression, and bl measurements were taken in the sagittal plane (Figure 1B,C). All diagnosticians scored their certainty in each of their measurements as “confident”, “halfway confident”, and “not confident”. All diagnosticians were blinded to the reference measurements. Diagnostician 1 (M.R.) also performed the reference measurements. However, there was more than one year between these and the CBCT measurements, so a memory effect can be ruled out.

Diagnostician 1 was already experienced in the measurement protocol from an earlier study [7]. Diagnostician 2 and diagnostician 3 performed measurements on two HD-CBCT scans of human mandibles and discussed the measurements with an experienced diagnostician until agreement was reached. The mandibles used for calibration were different from those used in this study.

Diagnostician 2 and diagnostician 3 performed the second measurements after a two-month break to avoid a memory effect. During this break, diagnostician 2 and diagnostician 3 received a single 20-min training session on LD-CBCT from diagnostician 1. In the training session, the diagnostician performed 10 LD-CBCT and HD-CBCT measurements according to the described methods of this study with diagnostician 1 and discussed the results until agreement was reached. The images taken during the training session were different to those used in the study.

### 2.4. Statistical Analysis

The measurement accuracy was evaluated as the absolute difference between the bone-loss measured using HD- or LD-CBCT and the reference bone-loss measurement obtained clinically. The reference bone-loss, measured bone-loss, and absolute difference between both were described numerically using quartiles, mean, and SD and graphically displayed using boxplots for all three diagnosticians. The measurement certainty of the diagnosticians was described using absolute and relative frequencies. All descriptive analyses were performed separately depending on the dose type (HD-CBCT, LD-CBCT) and measurement time (baseline, after training).

To investigate whether diagnosticians differed regarding bone-loss measurement accuracy at baseline, a Friedmann test was performed separately for each CBCT method. Upon rejection of the null hypothesis, pairwise comparisons of the diagnosticians were performed and the resulting *p*-values Holm-adjusted for type-I error inflation.

Moreover, a Wilcoxon two-sample signed rank test was used to compare the measurement accuracy of each diagnostician between baseline and after training for each CBCT method.

Further, the difference in measurement accuracy between baseline and after training was compared between diagnostician 2 and diagnostician 3 using a Wilcoxon two-sample signed-rank test. The goal was to assess whether training impacted measurement accuracy differently in diagnostician 2 and 3.

Assessing whether the diagnosticians’ experience was associated with their confidence in their measurements was performed separately for each CBCT method using a Pearson chi-squared test. For each diagnostician, it was also investigated whether training improved the confidence level in their measurements. Therefore, the association between the measurement time (baseline, after training) and the confidence level of the measurements was investigated using a Pearson chi-squared test. All analyses were performed using the statistical software R version 4.1.3 [13,14,15,16,17].

## 3. Results

The mean bone-loss reference measurement was 5.07 ± 1.2 mm, as shown in Table 1. The mean HD-CBCT and LD-CBCT measurements for diagnostician 1, 2, and 3 are shown in Table 1, Table 2 and Table 3, respectively. The mean HD- and LD-CBCT measurements for diagnostician 2 and 3 are shown separately for baseline and after training.

**Figure 2:**
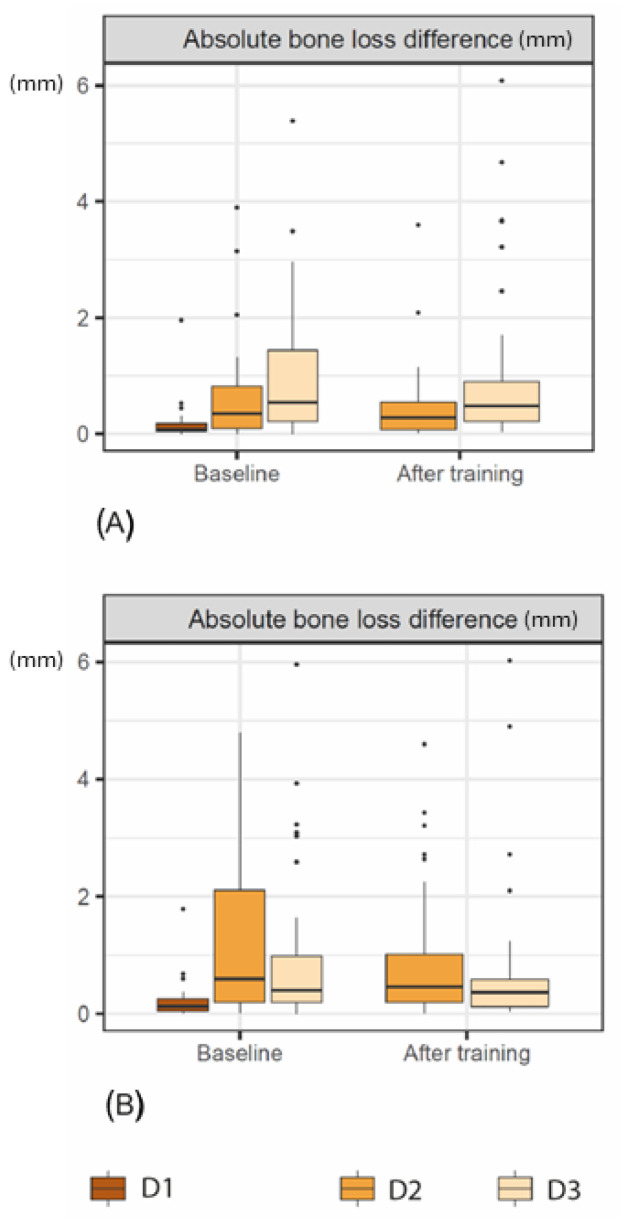
Boxplots of the raw and absolute bone-loss measurements (mm) at baseline and after training for each diagnostician (D1–D3) using (**A**) HD-CBCT and (**B**) LD-CBCT.

Regarding the baseline HD-CBCT measurements, there was a significant difference between diagnostician 1 and diagnostician 2 (*p* = 0.0042) and between diagnostician 1 and diagnostician 3 (*p* < 0.001), but not between diagnostician 2 and diagnostician 3 (*p* = 0.48). Similarly, for LD-CBCT, there was a significant difference between diagnostician 1 and diagnostician 2 (*p* < 0.001), diagnostician 1 and diagnostician 3 (*p* = 0.0012), but not between diagnostician 2 and diagnostician 3 (*p* = 0.563). Training slightly but not significantly improved the accuracy of HD-CBCT (*p* = 0.178) and LD-CBCT (*p* = 0.123) measurements in diagnostician 2 and LD-CBCT measurements (*p* = 0.897) in diagnostician 3 (see Figure 2 and Table 4).

Further, although not significantly (HD-CBCT: *p* = 0.428, LD-CBCT: *p* = 0.51), training improved the measurement accuracy of diagnostician 2 more than diagnostician 3 independently of the CBCT method used (see Table 5).

Regarding the certainty of diagnosticians in their measurements, there was a significant association between the diagnosticians’ experience and their confidence level before training for both HD-CBCT (*p* < 0.001) and LD-CBCT (*p* < 0.001) measurements (see Table 6).

Moreover, training improved certainty in diagnosticians 2 and 3 independently of the CBCT method, as shown in Table 7 and Table 8. For diagnostician 2, the observed improvement was not significant for both methods (HD-CBCT: *p* = 0.097; LD-CBCT: *p* = 0.326), whereas for diagnostician 3, it was significant (HD-CBCT: *p* < 0.001; LD-CBCT: *p* = 0.002).

## 4. Discussion

We proved the first hypotheses tested in this study, which is that examiner experience influences the measurements of the buccal lamella by HD-CBCT and LD-CBCT. Diagnostician 1, who had the most experience, showed significantly better results than diagnostician 2 and diagnostician 3 for both methods. This is in line with previous findings in the field of endodontology [11]. The accuracy of bone-loss measurements made by diagnostician 1 were consistent with the results of previous studies of HD-CBCT and LD-CBCT. It is therefore important that, to achieve repeatable and valid measurements in professional practice, this type of examination should be calibrated and relevant training provided.

There were no significant differences in the measurements made by the less experienced diagnosticians, and the mean absolute differences in measurements made by diagnosticians 2 and 3 compared with the reference measurements were 1.28 mm (±1.5) or less. After training, these values improved to 0.95 mm (±1.2) or less, which were in a clinically acceptable range. The Q1–Q3 values were also smaller after training, indicating an improvement in measurements and a positive training effect (Figure 1, Table 2 and Table 3). The individual differences in the training effect observed in diagnosticians 2 and 3 also indicate that the necessary number of training sessions depends on the individual investigator. However, this was to be expected. But, it is promising that measurement differences were similar for LD-CBCT and HD-CBCT, even among inexperienced diagnosticians, and that these values were within clinically acceptable limits.

Our findings also indicated that diagnostician experience plays a significant role in how certain diagnosticians are about their bone-loss measurements at baseline. The most experienced diagnostician was largely confident with their LD-CBCT and HD-CBCT measurements, whereas the less-experienced diagnosticians were never totally confident of their measurements. After a single training session, we observed a partial improvement in the certainly of diagnosticians 2 and 3 in their bone-loss measurements made by HD-CBCT and LD-CBCT. While the single training session significantly improved the subjective certainty of diagnostician 3 in their HD-CBCT and LD-CBCT measurements, the improvements observed in diagnostician 2 were not significant. These findings suggest that examiner experience has a stronger effect on certainty than the imaging modality does. Considering the diffusion and application of this technology, these topics should be explored and investigated in greater depth in future studies. The ex vivo nature of the experiments eliminated the risk of natural motion, such as tremors, which can lead to motion artifacts that can significantly reduce the quality and information content of the image [18,19]. Furthermore, none of the teeth investigated had extensive metallic or ceramic restorations, which can also cause imaging artifacts that can interfere with proper visualization of the edges of the teeth [19]. But usually, orthodontic patients are young and have only few fillings.

Only half heads were used. To mimic the missing half, gel pads were used as described in the Methods section. However, these pads cannot imitate natural bony structures, teeth, or restorative materials or the artifacts caused by these structures. Thus, the other half of the head cannot be fully accurately replicated. This means that the image quality may have been slightly better than it would have been for complete heads [20].

This study included only single-rooted teeth, but not molars. Segmentation may be more difficult due to the more complex anatomy. This fact may somewhat limit the generalizability of the results.

## 5. Conclusions

This study demonstrates that the examiner experience level plays an important role regarding the accuracy and certainty of bone-loss measurements when using HD- and LD-CBCT. For unexperienced or less experienced examiners, one short training session improved the accuracy and certainty of measurements. Accordingly, examiners should receive training before interpretating LD-CBCT images. However, the number of training sessions may vary depending on the investigator.

## Figures and Tables

**Table 1:** Bone-loss measurements for diagnostician 1 compared with reference measurements. HD = HD-CBCT, LD = LD-CBCT.

	Bl Clinical (mm)	Bl Measured (mm)		Absolute Bl Difference (mm)	
	*(reference)*	*HD*	*LD*	*HD*	*LD*
***n***	30	30	30	30	30
**Mean (±SD)**	5.07 (±1.2)	5.11 (±1.3)	5.14 (±1.25)	0.19 (±0.36)	0.23 (±0.34)
**Median**	4.8	4.75	4.78	0.08	0.12
**Q1–Q3**	4.2–6	3.95–6.03	4.09–6.05	0.05–0.2	0.05–0.26
**Min–Max**	3.2–7.4	3.32–7.51	3.33–7.37	0–1.96	0.01–1.79

**Table 2:** Bone-loss measurements for diagnostician 2 compared with reference measurements. bef. tr. = before training; aft. tr. = after training.

	Bl Measured (mm)				Absolute Bl Difference (mm)			
	*HD*		*LD*		*HD*		*LD*	
	*Bef. Tr.*	*Aft. Tr.*	*Bef. Tr.*	*Aft. Tr.*	*Bef. Tr.*	*Aft. Tr.*	*Bef. Tr.*	*Aft. Tr.*
*n*	30	30	30	30	30	30	30	30
**Mean (±SD)** **(mm)**	5.66 (±1.59)	5.38 (±1.68)	5.99 (±2.44)	5.84 (±2.08)	0.68 (±0.9)	0.48 (±0.72)	1.28 (±1.5)	0.95 (±1.2)
**Median** **(mm)**	5.4	4.68	5.08	5.2	0.36	0.28	0.59	0.46
**Q1–Q3** **(mm)**	4.23–6.55	4–6.36	4.1–7.49	4.06–7.32	0.09–0.87	0.07–0.56	0.19–2.29	0.18–1.03
**Min–Max** **(mm)**	3.74–10.5	3.25–10.8	2.94–11.5	3.57–11.8	0–3.9	0.02–3.6	0.01–4.8	0.01–4.6

**Table 3:** Bone-loss measurements for diagnostician 3 compared with reference measurements. bef. tr. = before training; aft. tr. = after training.

	Bl Measured (mm)				Absolute Bl Difference (mm)			
	*HD*		*LD*		*HD*		*LD*	
	*Bef. Tr.*	*Aft. Tr.*	*Bef. Tr.*	*Aft. Tr.*	*Bef. Tr.*	*Aft. Tr.*	*Bef. Tr.*	*Aft. Tr.*
***n***	30	30	30	30	30	30	30	30
**Mean (±SD)** **(mm)**	5.08 (±1.93)	5.48 (±2.23)	5.19 (±1.91)	4.75 (±1.79)	1.05 (±1.27)	1.15 (±1.56)	1.04 (±1.45)	0.84 (±1.4)
**Median** **(mm)**	4.5	4.49	4.53	4.31	0.55	0.49	0.4	0.36
**Q1–Q3** **(mm)**	3.82–6.13	3.85–7.1	4.04–6.27	3.58–6.04	0.2–1.54	−0.21–0.92	0.17–1.05	0.12–0.59
**Min–Max** **(mm)**	1.01–9.53	1.12–9.88	1.24–9.93	1.1–9.32	0–5.39	0.04–6.08	0–5.96	0.04–6.03

**Table 4:** Comparison of diagnostician bone-loss measurement accuracy between baseline and after training when using (A) HD-CBCT and (B) LD-CBCT.

**(A)**			
	**Baseline** **(*n* = 30)**	**After Training** **(*n* = 30)**	
	**Mean (±SD)**	**Mean (±SD)**	***p*-Value**
diagnostician 2	0.68 (±0.9)	0.48 (±0.72)	0.178
diagnostician 3	1.051 (±1.267)	1.147 (±1.558)	0.789
**(B)**			
	**Baseline** **(*n* = 30)**	**After training ** **(*n* = 30)**	
	**Mean (±SD)**	**Mean (±SD)**	***p*-Value**
diagnostician 2	1.278 (±1.503)	0.95 (±1.201)	0.123
diagnostician 3	1.041 (±1.451)	0.84 (±1.402)	0.897

**Table 5:** Comparison between bone-loss measurement accuracy improvement of the diagnosticians after training.

	Diagnostician 2 (*n* = 30)	Diagnostician 3 (*n* = 30)	
	Mean (±SD)	Mean (±SD)	*p*-Value
HD-CBCT	0.2 (±1.199)	−0.096 (±1.821)	0.428
LD-CBCT	0.33 (±1.039)	0.2 (±1.189)	0.51

**Table 6:** Baseline relationship between diagnosticians’ experience and certainty of bone-loss measurements using (A) HD-CBCT and (B) LD-CBCT.

**(A)**					
**Certainty Index (Baseline)**	**Diagnostician 1** **(*n* = 30)**	**Diagnostician 2** **(*n* = 30)**	**Diagnostician 3** **(*n* = 30)**	**Total** **(*n* = 90)**	***p***
not confident	0 (0%)	2 (7%)	10 (33%)	12 (13%)	<0.001
halfway confident	3 (10%)	16 (53%)	20 (67%)	39 (43%)	
confident	27 (90%)	12 (40%)	0 (0%)	39 (43%)	
**(B)**					
**Certainty Index (Baseline)**	**Diagnostician 1** **(*n* = 30)**	**Diagnostician 2** **(*n* = 30)**	**Diagnostician 3** **(*n* = 30)**	**Total** **(*n* = 90)**	***p***
not confident	0 (0%)	5 (17%)	8 (27%)	13 (14%)	<0.001
halfway confident	7 (23%)	18 (60%)	22 (73%)	47 (52%)	
confident	23 (77%)	7 (23%)	0 (0%)	30 (33%)	

**Table 7:** Relationship between training and certainty of bone-loss measurements of diagnostician 2 when using (A) HD-CBCT and (B) LD-CBCT.

**(A)**				
**Certainty Index (Baseline)**	**Baseline** **(*n* = 30)**	**After Training** **(*n* = 30)**	**Total** **(*n* = 60)**	***p***
not confident	2 (7%)	2 (7%)	4 (7%)	0.097
halfway confident	16 (53%)	8 (27%)	24 (40%)	
confident	12 (40%)	20 (67%)	32 (53%)	
**(B)**				
**Certainty Index (Baseline)**	**Baseline** **(*n* = 30)**	**After Training** **(*n* = 30)**	**Total** **(*n* = 60)**	***p***
not confident	5 (17%)	2 (7%)	7 (12%)	0.328
halfway confident	18 (60%)	23 (77%)	41 (68%)	
confident	7 (23%)	5 (17%)	12 (20%)	

**Table 8:** Relationship between training and certainty of bone-loss measurements of diagnostician 3 when using (A) HD-CBCT and (B) LD-CBCT.

**(A)**				
**Certainty Index (Baseline)**	**Baseline** **(*n* = 30)**	**After Training** **(*n* = 30)**	**Total** **(*n* = 60)**	***p***
not confident	10 (33%)	6 (20%)	16 (27%)	<0.001
halfway confident	20 (67%)	12 (40%)	32 (53%)	
confident	0 (67%)	12 (40%)	12 (20%)	
**(B)**				
**Certainty Index (Baseline)**	**Baseline** **(*n* = 30)**	**After Training** **(*n* = 30)**	**Total** **(*n* = 60)**	***p***
not confident	8 (27%)	5 (17%)	13 (22%)	0.002
halfway confident	22 (73%)	15 (50%)	37 (62%)	
confident	0 (0%)	10 (33%)	10 (17%)	

## Data Availability

The raw data supporting the conclusions of this article will be made available by the authors on request.

## References

[1] Fuhrmann A. (2013). Zahnärztliche Radiologie, Zmk Praxis.

[2] Hingst V., Weber M.A. (2020). Dentale Röntgendiagnostik Mit Der Panoramaschichtaufnahme—Technik Und Typische Bildbefunde. Der Radiologe.

[3] Ludlow J.B., Davies-Ludlow L.E., White S.C. (2008). Patient Risk Related to Common Dental Radiographic Examinations: The Impact of 2007 International Commission on Radiological Protection Recommendations Regarding Dose Calculation. J. Am. Dent. Assoc..

[4] Ludlow J.B., Timothy R., Walker C., Hunter R., Benavides E., Samuelson D.B., Scheske M.J. (2015). Effective Dose of Dental Cbct-a Meta Analysis of Published Data and Additional Data for Nine Cbct Units. Dentomaxillofac. Radiol..

[5] Cortellini P., Bissada N.F. (2018). Mucogingival Conditions in the Natural Dentition: Narrative Review, Case Definitions, and Diagnostic Considerations. J. Clin. Periodontol..

[6] Lee R.J., Weissheimer A., Pham J., Go L., de Menezes L.M., Redmond W.R., Loos J.F., Sameshima G.T., Tong H. (2015). Three-Dimensional Monitoring of Root Movement During Orthodontic Treatment. Am. J. Orthod. Dentofac. Orthop..

[7] Ruetters M., Gehrig H., Kronsteiner D., Doll S., Kim T.S., Lux C.J., Sen S. (2022). Low-Dose Cbct Imaging of Alveolar Buccal Bone Adjacent to Mandibular Anterior Teeth- a Pilot Study. Clin. Oral Investig..

[8] Ruetters M., Gehrig H., Kronsteiner D., Weyer V., Kim T.S., Lux C.J., Sen S. (2022). Ex-Vivo Imaging of Buccal and Oral Periodontal Bone with Low-Dose Cbct in Porcine Jaws. Dentomaxillofac. Radiol..

[9] Schwindling F.S., Hilgenfeld T., Weber D., Kosinski M.A., Rammelsberg P., Tasaka A. (2019). In Vitro Diagnostic Accuracy of Low-Dose Cbct for Evaluation of Peri-Implant Bone Lesions. Clin. Oral Implants Res..

[10] Ruetters M., Gehrig H., Kim T.S., Bartha V., Bruckner T., Schwindling F.S., Felten A., Lux C., Sen S. (2022). Imaging Furcation Defects with Low-Dose Cone Beam Computed Tomography. Sci. Rep..

[11] Parker J.M., Mol A., Rivera E.M., Tawil P.Z. (2017). Cone-Beam Computed Tomography Uses in Clinical Endodontics: Observer Variability in Detecting Periapical Lesions. J. Endod..

[12] R Core Team (2021). A Language and Environment for Statistical Computing.

[13] Gamer M., Lemon J., Singh I.F.P. (2012). Irr: Various Coefficients of Interrater Reliability and Agreement.

[14] Signorell A. (2020). Desctools: Tools for Descriptive Statistics.

[15] Signorell A., Aho K., Alfons A., Anderegg N., Aragon T., Arppe A., Baddeley A., Barton K., Bolker B., Borchers H.W. (2019). Desctools: Tools for Descriptive Statistics.

[16] Lehnert B. (2015). Blandaltmanleh: Plots (Slightly Extended) Bland-Altman Plots.

[17] Brüllmann D., Schulze R.K.W. (2015). Spatial Resolution in Cbct Machines for Dental/Maxillofacial Applications—What Do We Know Today?. Dentomaxillofac. Radiol..

[18] Kiljunen T., Kaasalainen T., Suomalainen A., Kortesniemi M. (2015). Dental Cone Beam Ct: A Review. Phys. Medica.

[19] Schulze R., Heil U., Gross D., Bruellmann D.D., Dranischnikow E., Schwanecke U., Schoemer E. (2011). Artefacts in Cbct: A Review. Dentomaxillofac. Radiol..

[20] McGarry C.K., Grattan L.J., Ivory A.M., Leek F., Liney G.P., Liu Y., Miloro P., Rai R., Robinson A., Shih A.J. (2020). Tissue Mimicking Materials for Imaging and Therapy Phantoms: A Review. Phys. Med. Biol..

